# Use of supplementary phenotype to identify additional rheumatoid arthritis loci in a linkage analysis of 342 UK affected sibling pair families

**DOI:** 10.1186/1471-2350-10-142

**Published:** 2009-12-21

**Authors:** Bamidele O Tayo, Yulan Liang, Arpad Kelemen, Austin Miller, Maurizio Trevisan, Richard S Cooper

**Affiliations:** 1 Department of Preventive Medicine and Epidemiology, Loyola University Chicago, Chicago, IL, USA; 2 Department of Family and Community Health, University of Maryland, Baltimore, MD, USA; 3 Department of Organizational System and Adult Health, University of Maryland, Baltimore, MD, USA; 4 Departments of Biostatistics, State University of New York at Buffalo, Buffalo, NY, USA; 5 University of Nevada Health Sciences System, Las Vegas, NV, USA

## Abstract

**Background:**

Although rheumatoid arthritis has been shown to have moderately strong genetic component, both linked loci identified in linkage analyses and susceptibility variants from association studies are short of adequately accounting for a comprehensive catalogue of the molecular factors underlying this complex disease. The objective of this study was to use supplementary phenotype based on cumulative hazard of rheumatoid arthritis to identify linkage evidence for new and additional rheumatoid arthritis loci in a genome-wide linkage analysis of 342 affected sibling pair families from the United Kingdom.

**Methods:**

Using proportional hazards model, we estimated cumulative hazard of rheumatoid arthritis and then used it as a quantitative trait in a non-parametric multipoint variance component linkage analysis with 353 microsatellite markers distributed across the 22 autosomal chromosomes.

**Results:**

We identified 3 new loci with genome-wide suggestive linkage evidence for rheumatoid arthritis on 9q21.13, 15p11.1 and 20q13.33. Our results also confirmed previously reported linkage evidence in the HLA-DRB1 region on chromosome 6 and on locus 1q32.1.

**Conclusion:**

This study demonstrates the potential for information gain through the use of supplementary phenotypes in genetic study of complex diseases to identify new and additional potential linked loci that are not detected by linkage analysis of traditional phenotypes; and our results provide further evidence of the involvement of multiple loci in the genetic aetiology of rheumatoid arthritis.

## Background

Rheumatoid arthritis (RA) is a chronic inflammatory condition that belongs to the family of diseases referred to as autoimmune. The population prevalence of RA is about 0.01 [1,2], and the relative risk for siblings of affected persons ranges between 2 and 17 [2-5]. In addition to evidence from familial aggregation, results from twin studies [6-8], segregation analysis [9] and genome-wide association studies [10-12] all indicate evidence of genetic influence in the aetiology of RA. This evidence is further strengthened by the relatively high heritability of RA, estimated to be about 65% [13] from twin studies; and also by reported linkage evidence for regions on chromosomes 2 and 11 [14], and 6 and 16 [15]. Although consistently strong evidence across studies points to association with the HLA locus on chromosome 6, it has however been observed that the RA-HLA region association accounts for only about one-third of the total genetic determinants of susceptibility to RA [16,17]. On the other hand, none of the other RA-linked loci reported to date has been consistently reported across studies [1,3,14,18,19]. The implication is that it is likely that a number of other loci involved in the genetic susceptibility to RA exist and are yet to be identified and their individual roles quantified; hence the complete role of genetic factor in the aetiology of this complex disease remains to be clearly understood.

As with many other complex human diseases, the difficulty in achieving a clear understanding of the role of genetics in disease can partly be attributed to limitations in phenotype definition and characterization. RA is a complex disease condition with variation in onset age, severity, and accompanying presence or absence of clinical features such as nodules, erosions, and rheumatoid factor. In addition, phenotypic, clinical and genetic heterogeneity exists between studies. To address the problem of heterogeneity associated with RA phenotype, John et al. [15] and Eyre et al. [20] used stratified analyses based on subgroups defined by age at disease onset, sex and clinical features such as rheumatoid factor (RF) status, erosion and shared epitopes. In addition to the affection status variable, age at RA onset provides the opportunity to investigate genetic influence based on the cumulative hazard of RA by using survival analysis tool in combination with linkage analysis. The premise is that detecting evidence of linkage in regions not previously identified in analysis of affection-based phenotype may be possible in analysis of supplementary phenotype of the same disease. The objective of this study was to use cumulative hazard estimates from proportional hazards model in genome-wide scan for linkage evidence for new and additional genetic loci linked to RA using affected sibling pairs data. We present here the results from the genome-wide linkage analysis of the cumulative hazard to RA in a sample of 728 relatives from 342 affected sibling pair families from the UK.

## Methods

The RA affected sibling pair families used in this study were part of the UK National Repository of Multicase Rheumatoid Arthritis Families maintained by the Arthritis Research Campaign (http://www.medicine.manchester.ac.uk/epidemiology/research/arc/). Details of family recruitment and assessment protocols have been described elsewhere [1,20]. Briefly, subjects were examined by trained metrologists using standardized questionnaires and protocols. Radiographs of each subject's hands were reviewed and rheumatoid factor status of each subject was ascertained using a particle agglutination test. All RA affected subjects satisfied the American College of Rheumatology criteria [21,22]. Access to the UK RA data was requested and approved through the Genetic Analysis Workshop 15 (GAW15). The data used in the present study were from cohorts 1 and 2 families as made available through GAW15 [23]. Cohort 1 comprised of RA affected sibling pairs from the 182 families as described by Mackay *et al *[1] and cohort 2 was made up of RA affected sibling pairs from the additional 195 families which have been described elsewhere [20]. Available phenotypic and clinical information included RA affection status, age at onset of RA symptoms, erosion status, and RF status. Genotype data on 353 informative microsatellite markers from the ABI Prism Linkage Mapping set, spanning the 22 autosomes at an average inter-marker distance of 10 cM were available on subjects and used in the present study.

### Estimation of cumulative hazard

We restricted all analyses to RA affection status, age at onset and sex since other measures such as severity of RA, erosion status and RF status are measures of post-RA affection symptoms. Our objective was to study only those variables that influence an individual's hazard to RA. Survival analysis was performed using RA status, sex, and onset age data on 750 affected subjects from 364 families. Since first symptoms of RA can begin at any point in time, it is reasonable to suppose that observed ties in event times (age at onset) among subjects are likely results of approximation or convenience in measurement and that there is a true but unknown ordering for the tied event times. This supposition appears more plausible for event as onset of RA and as such tied event times were treated according to the method described by Kalbfleisch and Prentice [24], and DeLong and colleagues [25]. Briefly, in the fitted proportional hazards model, exact conditional probability was computed for each event under the assumption that all tied event times occur before censored times of the same value or before larger values. The proportional hazards model was based on the assumption that the hazard to RA for any subject is a fixed proportion of the hazard to RA for any other subject in the study sample and the ratios of hazards are independent of time. The use of Cox regression to fit the proportional hazards model in this study allowed us not to specify any particular distribution for the underlying hazard function and also to test the hypothesis of proportionality of hazards. We included sex as a covariate and then modelled the survival function from which the cumulative hazard for each subject was estimated as the negative of the logarithm of the survival function as -log*S*(*t*); where *S*(*t*) is the survival function at time 't' estimated from the fitted proportional hazards model.

### Linkage analysis

For the linkage analysis, 22 families with singletons were excluded because these were uninformative for linkage analysis. The linkage analysis was therefore based on 342 families with a total of 728 affected siblings. There were 353 microsatellite markers across the 22 autosomal chromosomes. For subjects not genotyped for some markers, their genotypes were set to missing for such markers. Allele frequencies for each marker were calculated using maximum likelihood estimation method. The marker-specific identity by descent (IBD) for sibling pairs within each family, and the multipoint IBDs were computed using the software SOLAR [26]. Since the estimated cumulative hazard was on continuous scale, we therefore used it as a quantitative trait in the linkage analysis as opposed to a dichotomous trait that is used when RA is analysed as affection status. The computed multipoint IBDs were used in a nonparametric multipoint linkage analysis of the cumulative hazard of RA using the software SOLAR. The use of nonparametric linkage method enabled us not to specify any inheritance model since the aetiology of RA is complex. Linkage evidence was expressed as logarithm of odds (LOD) scores for linkage at each chromosomal position. LOD scores which were defined as the logarithm to base 10 of likelihood ratio of the data assuming linkage versus the analysis model assuming no linkage, were used to summarize the evidence in favour of linkage and these plotted by chromosome against the genetic marker positions.

## Results

The distributions of pairs of RA affected siblings and sexes in the total sample of 342 families included in the linkage analysis are shown in Table 1. There were 306 families with 2 affected siblings each, 31 families with 3 affected siblings each, and 5 families with more than 3 affected siblings each. The 728 subjects comprised of 537 females and 191 males. Mean age in years at onset of RA is 41.6 for the sample, 41.4 for females and 42.2 for males (*P *= 0.8360) (Table 1). Influence of gender on RA hazard was assessed by including sex as a covariate in the proportional hazards model and then testing the null hypothesis that its coefficient is not different from zero. We also tested the hypothesis of proportionality of hazards in our study sample. For both tests, we did not observe any statistical evidence against the null hypotheses of proportionality of hazards and that of equality between zero and coefficient of sex. The *p*-value for the coefficient of sex was 0.5109 and the *p*-value for the Kolmogorov-type supremum test for proportional hazards assumption was 0.9640 based on 1000 bootstrap samples.

**Table 1:** Distributions of affected sibling pair sizes

Size of sibling pair	No. of families	No. of subjects	Mean onset age (years ± standard error)
2	306	612	-
3	31	93	-
4	3	12	-
5	1	5	-
6	1	6	-
All	342	728	41.63 ± 0.51
Females	-	537	41.44 ± 0.61
Males	-	191	42.17 ± 0.89

**Figure 1:**
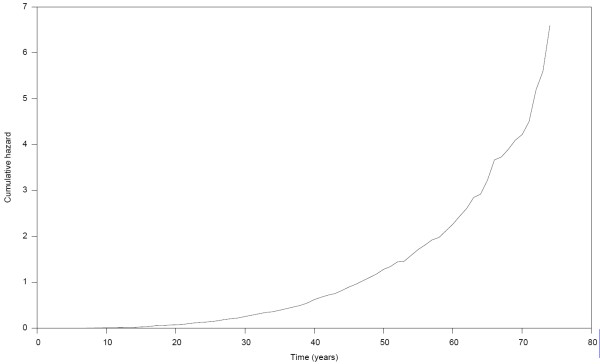
**Distribution of cumulative hazard of rheumatoid arthritis**.

**Figure 2:**
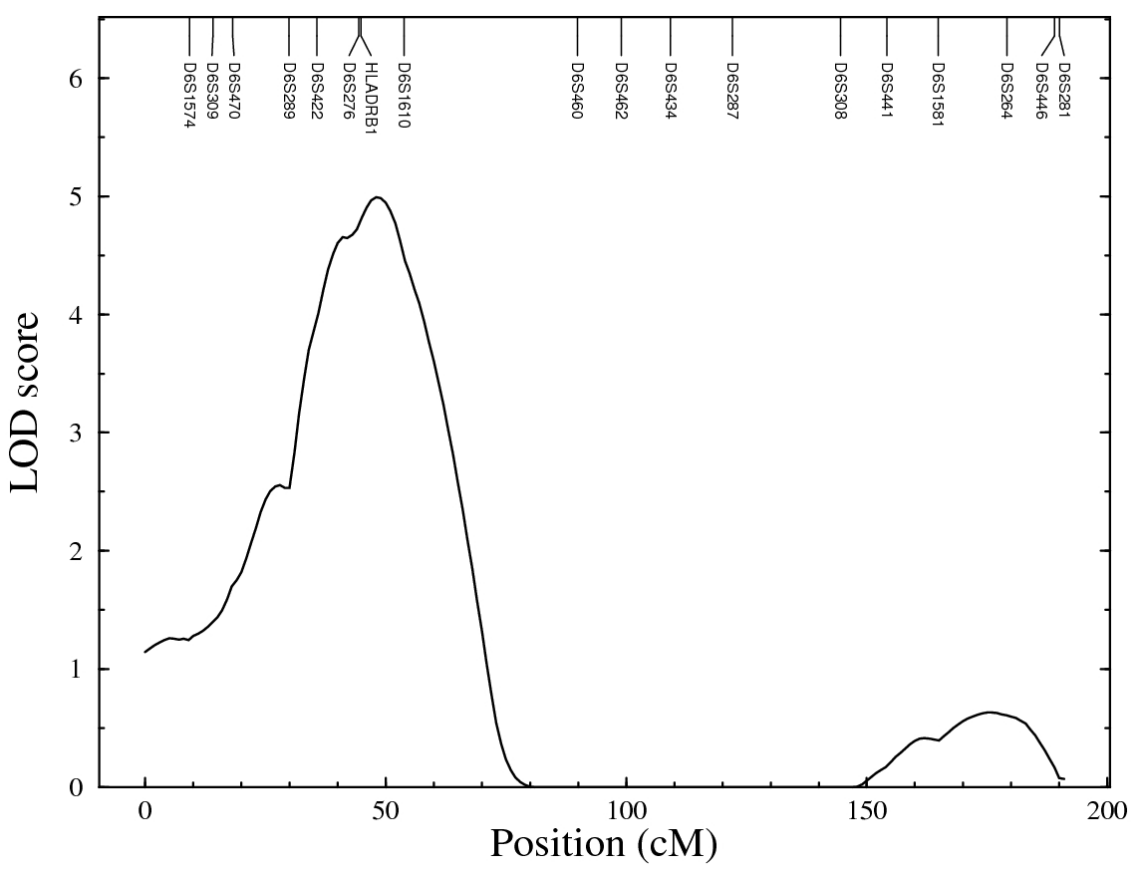
**Region of significant genome-wide linkage evidence on chromosome 6**.

**Figure 3:**
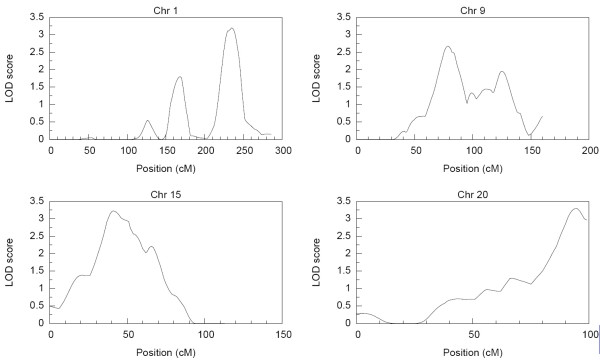
**Chromosomal regions with suggestive linkage evidence**.

**Figure 4:**
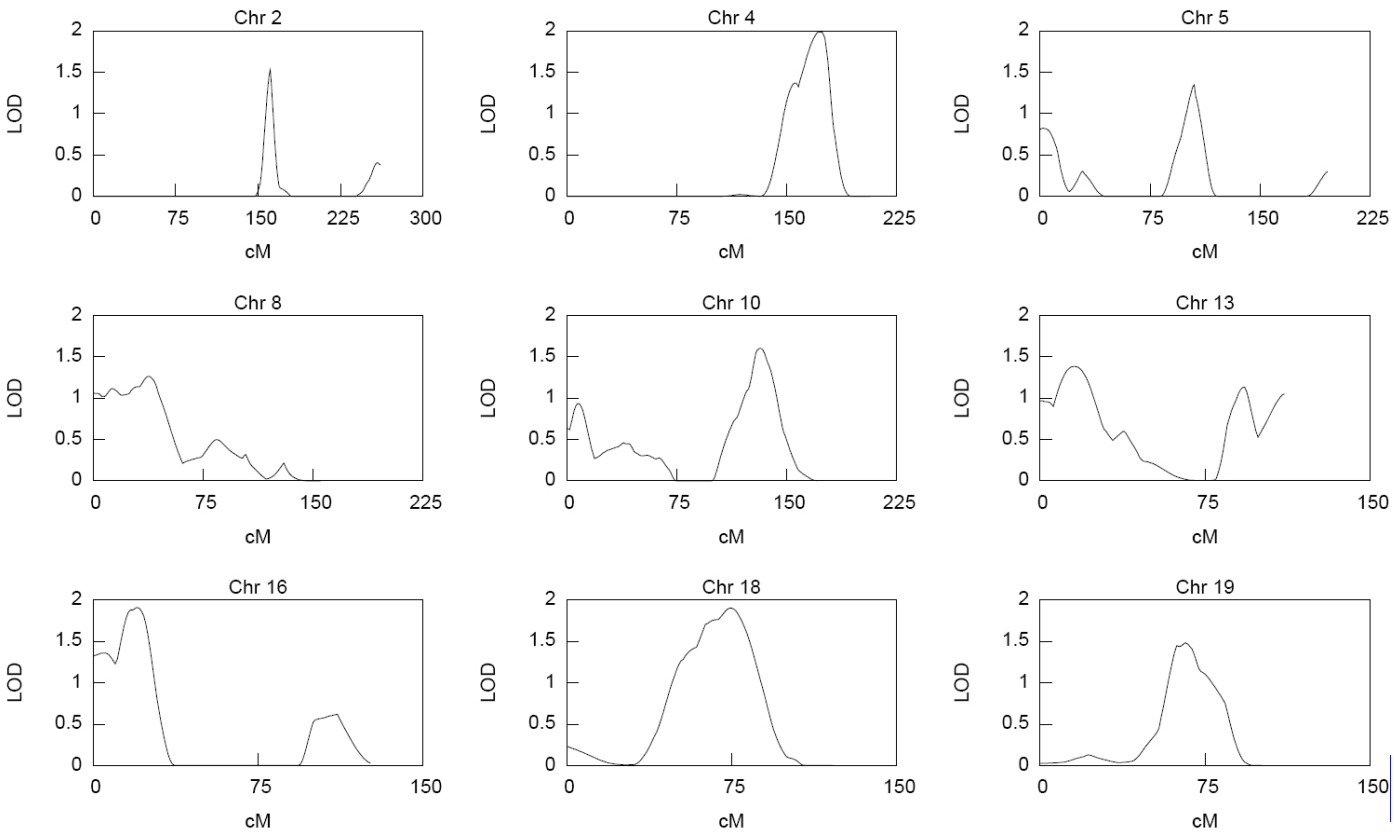
**Selected chromosomal regions with nominal linkage evidence**.

The distribution of the estimated cumulative hazard function is presented in Figure 1. The result shows a rather slow rise in cumulative hazard from the early years up till about age 42 years, which is also about the median survival time, and thereafter the cumulative hazard began to accelerate more rapidly with increasing years. The strongest linkage evidence observed on each chromosome from the results of the nonparametric multipoint linkage analysis using the 353 microsatellites markers are presented in Table 2. Based on generally accepted thresholds of LOD scores for genome-wide linkage studies [27], we observed genome-wide significant evidence (LOD score ≥ 3.6; *P *≤ 2 × 10^-5^) for linkage on chromosome 6. The linkage signal spanned the HLA-DRB1 region (Figure 2) which has been reported in other studies [1-3,14,18,28,29] to contain potential RA loci. Other chromosomal regions with genome-wide suggestive evidence for linkage (LOD score ≥ 2.2; *P *≤ 7.4 × 10^-4^) were identified on chromosomes 1, 9, 15 and 20 and are displayed in Figure 3. The LOD score of 3.3 on chromosome 20 at about 94 cM near the D20S173 was the second highest followed by the LOD score of 3.2 on chromosome 15 near the D15S994 marker at about 41 cM. The signal on chromosome 1 was observed in the region previously identified in other reports [1,18,20,28-30], whereas those on chromosomes 9, 15 and 20 have not previously been reported and thus represent newly identified potential RA loci. The lengths of the corresponding 1-LOD support intervals for the linkage evidence on chromosomes 1, 6 and 15 were 22 cM, 31 cM and 26 cM, respectively. The location of the highest linkage signal on chromosome 20 was near the end of the chromosome thus making a reliable estimation of its support interval difficult. Genome-wide nominal linkage evidence (LOD ≥ 0.8; *P *< 0.05) was identified on eleven other chromosomal regions (Figure 4), some of these corresponded to previously reported regions with similar level of linkage signal for RA [1,20,30].

**Table 2:** Regions with highest LOD scores on each chromosome

Chromosome	Locus	LOD score	Position (cM)‡	Nearest marker
1	1q32.1	3.2	235	D1S425
2	2q24.1	1.5	161	D2S142
3	3p26.3	1.0	8	D3S1297
4	4q34.1	2.0	173	D4S1539
5	5q15	1.3	105	D5S644
6	6p21.3	5.0	48	HLA-DRB1
7	7q36.2	0.5	181	D7S2465
8	8q21.3	1.3	38	D8S258
9	9q21.13	2.7	78	D9S167
10	10q25.1	1.6	132	D10S597
11	11q	0.1	86	D11S937
12	12q24.33	1.0	165	D12S1723
13	13q12.3	1.4	15	D13S217
14	14q13	0.6	57	D14S276
15	15p11.1	3.2	41	D15S994
16	16q13.13	1.9	20	D16S404
17	-	0.8	104	D17S1790
18	18q21.1	1.9	74	D18S474
19	19q13.2	1.5	66	D19S420
20	20q13.33	3.3	94	D20S173
21	-	0.0	-	-
22	22q13.2	0.2	52	D22S274

‡cM: centiMorgan on Marshfield map

## Discussion

We report here genome-wide linkage evidence for RA based on analysis of cumulative hazard estimated from family data on RA affected sibling pairs. The objective of the study was to identify additional evidence of linkage for RA by using supplementary RA phenotype that we envisioned would capture linkage information otherwise undetected by the traditional affection-status phenotype definition used in previous linkage studies of RA. The supplementary phenotype approach was based on application of proportional hazards modelling to estimate cumulative hazard of RA based on affection status and RA onset age for each study subject. 750 affected subjects from 364 families were included in the estimation of cumulative hazard using proportional hazards model. The estimated cumulative hazard was then used as quantitative trait in linkage analysis of genotypes on 353 microsatellite markers spanning the 22 autosomes at an average inter-marker distance of 10 cM.

In this study we have identified three new potential RA loci which have not been reported or detected in previous linkage analyses that used the traditional RA affection status. The new potential RA loci with strong genome-wide suggestive linkage evidence are 9q21.13 with LOD score of 2.7, 15p11.1 with LOD score of 3.2 and 20q13.33 with LOD score of 3.3. In addition to the identified new potential RA loci, we also obtained linkage evidence in chromosomal regions that are consistent with previous findings. The most significant genome-wide linkage evidence was detected on chromosome 6 in the HLA-DRB1 region which is consistent with previous reports [1-3,14,18,28,29]. The genome-wide suggestive linkage evidence detected in 1q32.1 is also similar to reported level of linkage evidence in previous studies for the region [30]. Among the candidate genes located in locus 1q32.1 are CHI3L1 (chitinase 3-like 1 (cartilage glycoprotein-39)) gene which has been implicated in local autoimmune response that leads to chronic inflammation and joint destruction in RA patients [31] and the IKBKE (inhibitor of kappa light polypeptide gene enhancer in B-cells, kinase epsilon) gene which has been reported to play a role in synovial inflammation, extracellular matrix destruction, and activation of the viral program and innate immune response in RA [32]. Also, the HRH3 (histamine receptor H3) gene located in locus 20q13.33, has a role in neurogenic inflammation [33].

We note that in a previous linkage analysis of a subset of these data (cohort 1 families), Mackay et al [1] reported significant genome-wide evidence on chromosome 6 in the same HLA-DRB1 region on which we have also detected significant evidence, and also identified a region on same chromosome with suggestive evidence plus 9 regions of nominal evidence on other chromosomes. Likewise, in a slightly larger sample from the same UK cohort 1 families, John et al [15] confirmed the linkage evidence on chromosome 6 previously reported by Mackay et al [1], but did not detect any suggestive linkage evidence outside of chromosome 6. However, in another linkage study involving both cohorts 1 and 2 families, Eyre et al [20] failed to detect any significant or suggestive linkage evidence. The fact that previously reported potential RA loci are replicated in the present study underscore the validity of the supplementary RA phenotype approach used here. The identification of new potential RA loci in this study is a demonstration of the importance of the use of supplementary phenotypes for genetic mapping of complex diseases such as RA.

It is worth noting that in this study we did not include clinical covariates such as rheumatoid factor status and erosion status that were included in some of the previous studies because these covariates measure symptoms resulting from RA affection. It is therefore not illogical to assume that an individual's cumulative hazard to RA could not be influenced by such post-RA onset phenotypes or clinical symptoms. We therefore chose not to include in the model variables that we think do not influence an individual's hazard of being affected by RA. Whether all the identified linkage regions in the present study are true positives requires further studies. We declare that the reproduction of linkage evidence at previously reported potential RA loci provides support for the validity of the definition of supplementary RA phenotype and modelling that we have used in the present study. We recognise that supplementary phenotype as defined and treated as quantitative trait in the present study may not be practicable for every complex human disease phenotype. However, for complex diseases for which this approach is applicable, this can lead to identification of new additional genetic loci that could explain part of the missing heritability [34] observed in susceptibility variants identified in association studies of complex diseases. This supplementary phenotype approach can be used to identify new candidate loci or narrow candidate regions in linkage and association studies. It is envisioned that results from both analyses of supplementary disease phenotypes and their corresponding traditional disease phenotype definitions would enhance the efficiency of the "genetic dragnet" at capturing important genetic variants contributing to complex diseases.

## Conclusion

This present study demonstrates the potential information gain through the use of supplementary phenotypes in genetic study of complex diseases such as RA to detect new and additional linked loci that are undetected in linkage analysis of the traditional phenotypes. Also, our results provide further evidence of the involvement of multiple loci in the genetic aetiology of RA.

## Competing interests

The authors declare that they have no competing interests.

## Authors' contributions

BOT conceived of the study, performed all statistical analyses, interpreted results and wrote the manuscript. YL participated in the design, statistical analyses, and preparation of manuscript. AK, AM, MT and RSC participated in the design and preparation of manuscript. All authors read and approved the final manuscript.

## Pre-publication history

The pre-publication history for this paper can be accessed here:

http://www.biomedcentral.com/1471-2350/10/142/prepub
